# The potential roles of gut microbiome in anal fistula

**DOI:** 10.1186/s13568-023-01560-9

**Published:** 2023-06-10

**Authors:** Ping Cai, Hao Rong, Qiaoqiao Zhu, Xiaoyu Dai, Jianpei Zhao

**Affiliations:** 1 Ningbo No.2 Hospital, Ningbo, 315000 China; 2 Ningbo Institute of Life and Health Industry, University of Chinese Academy of Sciences, Ningbo, 315000 China; 3 Key Laboratory of Diagnosis and Treatment of Digestive System Tumors of Zhejiang Province, Ningbo, 315000 China; 4 The Affiliated Hospital of Medical School of Ningbo University, Ningbo, 315000 China; 5 Department of Preventive Medicine, Zhejiang Provincial Key Laboratory of Pathological and Physiological Technology, School of Medicine, Ningbo, 315211 China

**Keywords:** Anal fistula, Gut microbiome, 16S rRNA gene sequencing, Clinical research

## Abstract

Anal fistula is a common proctological disease, but the thorough mechanisms of the anal fistula formation are still unclear. An increasing number of studies have revealed the crucial role of gut microbiota in intestinal diseases. We used 16S rRNA gene sequencing to analyze the intestinal microbiome in order to determine whether there are differences in the microbiome between anal fistula patients and healthy individuals. The microbiome samples were extracted by repeatedly wiping the rectal wall with intestinal swab. Before this operation, the whole intestine of all participants was irrigated and the score of the Boston bowel preparation scale reached 9. The biodiversity of gut microbiome of rectum revealed significant difference between anal fistula patients and healthy individuals. 36 discriminative taxa were identified by LEfSe analysis between two groups. At the phylum level, *Synergistetes* was enriched in anal fistula patients, while *Proteobacteria* was higher in healthy individuals. We also found that at the genus level, *Blautia*, *Faecalibacterium*, *Ruminococcus*, *Coprococcus*, *Bacteroides*, *Clostridium*, *Megamonas* and *Anaerotruncus* were highly enriched in anal fistula patients, while the microbiome of healthy individuals was enriched with *Peptoniphilus* and *Corynebacterium*. Spearman correlations showed the extensive and close association among genera and species. Finally, a diagnostic prediction model was constructed by random forest classifier, and the area under curve (AUC) reached 0.990. This study gave an important hint for analyzing gut microbiome of rectum in anal fistula patient.

Keypoints.

We use the 16S rRNA gene sequencing to test the microbiome samples extracted from the intestinal swab. This is the first study to explore the gut microbiome of rectum using this workflow. We also found the distinct gut microbiome of rectum differences between anal fistula patients and healthy individuals.

**Supplementary Information:**

The online version contains supplementary material available at 10.1186/s13568-023-01560-9.

## Introduction

Anal fistula is a common proctological disease seen by the anorectal surgeons. The incidence in male reached 12.3/100000 and the prevalence in female was approximately 5.6/100000 (Mei et al. 2019). Although great advances have been achieved in controlling and eradicating the fistula and preserving the anal continence (Joy and Williams 2002; Vial et al. 2010), such as fistulotomy (Sun et al. 2019), fistulectomy (Seyfried et al. 2018), cutting seton (Patton et al. 2015), ligation of the intersphincteric fistula tract (Xu and Tang 2017) and drainage seton (Daodu et al. 2018), the thorough mechanisms of the anal fistula formation are still unclear.

Nowadays, there is a widely accepted doctrine of the cryptoglandular theory proposed by Parks, which suggested that the infection of anal crypt gland leads to the anal fistula (Gosselink et al. 2015; Rizzo et al. 2010). However, this theory cannot explain the whole aspects of anal fistula. For example, a retrospective cohort study involved 148 patients with perianal abscesses found that the cumulative incidence of anal fistula was only 37% (Hamadani et al. 2009). In addition, the intestines of healthy individuals also have many detrimental bacteria strains that do not result in an anal fistula (Abbas et al. 2008; Gosselink et al. 2015).

An increasing number of studies have shown that the intestinal microbiome plays important roles in maintaining intestinal homeostasis (Haac et al. 2019). The disruption of the balance of the gut microbiome can lead to the intestinal diseases like colorectal cancer (Brennan and Garrett 2016) and inflammatory bowel diseases (Frank et al. 2007). For example, in the human colorectal cancer, the *Fusobacterium nucleatum*, *Bacteroides fragilis*, and *Escherichia coli* were considered to promote the tumorigenesis (Brennan and Garrett 2016). What’s more, increase in detrimental strains of *Proteobacteria* but decrease in the protective gut commensal strains of *Firmicutes* in patients with inflammatory bowel diseases compared to healthy individuals (Frank et al. 2007; Matsuoka and Kanai 2015). The evidences mentioned above reveal the key role of gut microbiome in intestinal diseases. This also raises a question of whether the gut microbiome of patients with anal fistula differs from that of healthy individuals, thus contributing to the formation of anal fistula? (Sugrue et al. 2017).

Here, we collected the microbiome samples from patients with anal fistula and healthy individuals. The 16S rRNA gene sequencing was performed to profile the intestinal microbiome. The analysis of Alpha and Beta diversities were performed to determine if the microbiome differs between anal fistula patients and healthy individuals. Finally, a diagnostic prediction model with the relative abundance of gut microbiome of rectum was established and its performance was tested. To our knowledge, it is the first study to explore the distinct gut microbiome of rectum differences between anal healthy individuals. Our results may provide new clues for the roles of gut microbiome of rectum in anal fistula patient.

## Methods

### Recruitment of individuals

In order to improve the accuracy and minimize the confounding factors, the exclusion criteria were established: history of chronic diseases like diabetes, hypertension and cancers, use of antibiotics in past 4 weeks, history of smoking or drinking. Ultimately, a total of 50 anal fistula patients and 50 healthy individuals were recruited at Hua Mei Hospital, University of Chinese Academy of Sciences. The anal fistula patients were diagnosed by digital rectal examination and nuclear magnetic resonance imaging.

Every participant agreed to take part in this project and the research was approved by the ethics committee of Hua Mei Hospital, University of Chinese Academy of Sciences (PJ-NBEY-KY-2020-042-01).

### Sample collection and DNA extraction

All participants underwent colonoscopy to rule out other intestinal diseases. Before the examination, the whole intestine was irrigated and the score of the Boston bowel preparation scale reached 9 (Lai et al. 2009). The microbiome samples were extracted by repeatedly wiping the rectal wall with intestinal swab. The samples were stored immediately at -80 ℃ for DNA extraction. The genome DNA was extracted by QIAGEN DNeasy PowerSoil Pro Kit. The library products were purified with SIVortex-Genie 2 and QIAGEN Vortex. The purity and concentration of products were checked by NanoDrop.

### 16S rRNA gene sequencing

The V3-V4 region of 16S ribosomal RNA gene in bacteria were amplified by PCR using primers 341F 5’-ACTCCTACGGGAGGCAGCA-3’ and 806R 5’-GGACTACHVGGGTWTCTAAT-3’. The workflow of PCR was shown as follow: (1) 30 cycles at 98 ℃ for 1 min, 98 ℃ for 10 s, 50 ℃ for 30 s, 72 ℃ for 1 min; (2) an extension at 72 ℃ for 5 min; (3) 1% agarose gels were used to monitor the products of PCR; (4) the PCR products were purified by DNA Clean Beads. Finally, the sequencing was performed on an Illumina MiSeq platform (Illumina, San Diego, CA, USA) with 250 bp paired-end reads.

### Bioinformatics analysis

The high-quality clean data were obtained using Qiime2 DADA2 function from raw data (Bokulich et al. 2013). Quality assessment of clean data were performed by FastQC (version 0.11.9). The analysis of Alpha and Beta diversities were performed using Qiime2 (version 2021.4.0). Naive bayes algorithm was used to cluster the bacteria into different operational taxonomic units (OTU) with 99% consistency. The OTU sequences were downloaded from the greengenes database to annotate microbiota. Linear discriminant analysis (LDA) effect size (LEfSe) was performed to identify the differential microbiota between two groups, and the significant level was served as *p-value* < 0.05 and the LDA score > 4. The random forest model was used to explore the diagnostic bacterial biomarkers by the “randomForest” package. The average the area under the receiver operating characteristic (ROC) curve value was used to evaluate the prediction model by the “pROC” package (Robin et al. 2011). The values of the probability of disease (POD) calculated by randomly generated decision trees were also used to evaluate the models. R version 4.1.2 was used to analyze the general data of participants. The differences between two groups in 16S rRNA gene were compared by the Wilcoxon signed-rank test. *P-value* < 0.05 was considered as significant level.

## Results

### Biodiversity of gut microbiome

100 participants including 50 anal fistula patients and 50 healthy individuals were recruited in our study. The demographic characteristics were shown in Table 1. We found that there were no significantly difference in age (*p-value* = 0.400) and sex (*p-value* = 0.342) between two groups.

**Table 1:** The general characteristics of anal fistula patients and healthy individuals

Characteristics	Healthy individuals (n = 50)	Anal fistula patients (n = 50)	*P-*value
Age (y)	41.68 ± 10.99	40.1 ± 12.0	0.400
Sex (male)	36/50 (72%)	41/50 (82%)	0.342

**Fig. 1:**
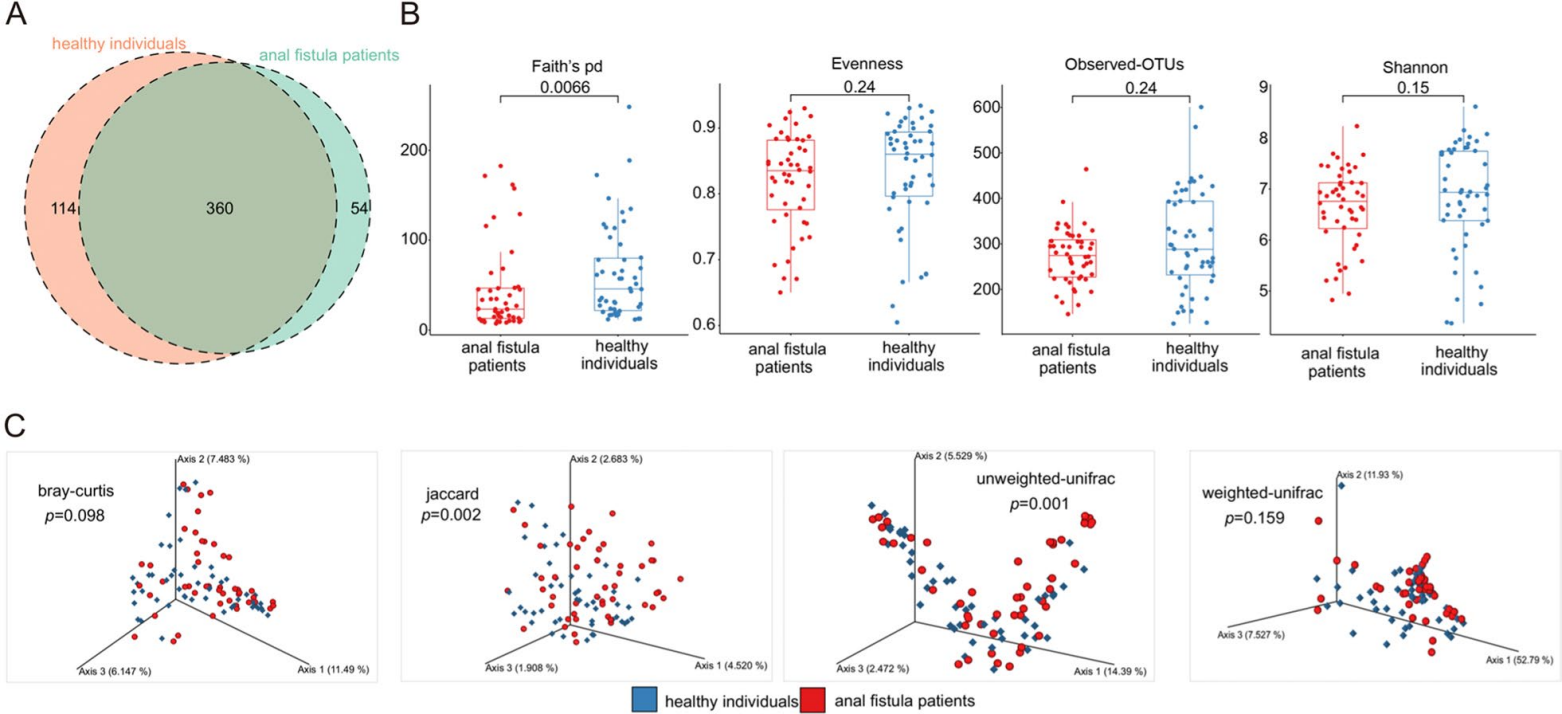
Biodiversity of gut microbiome between anal fistula patients and healthy individuals. **A** The numbers of OTUs in anal fistula patients and healthy individuals are shown in the venn diagram. **B** Barplots show the comparison of alpha diversity based on Evenness, Faith’s PD, observed-OTUs and Shannon between two groups. **C** 3D-PCoA plot shows the comparison of beta diversity based on Bray–Curtis distance, Jaccard distance, Unweighted Unifrac distance and Weighted Unifrac distance

After quality assurance, data from 2 anal fistula patients were excluded (the raw reads were too short) and the other 98 had data of optimal quality. We obtained 16589784 raw reads from 98 participants’ microbiome samples. Through the quality control, 14205383 clean reads were obtained with an average of 144953 reads per sample, and the average efficiency rate was 85.57% (Additional file 1: Table S1). These reads identified a total of 528 unique OTUs who were achieved with 99% consistency. Out of 528 OTUs, there were 54 unique OTUs in anal fistula patients, 114 unique OTUs in healthy individuals and 360 common OTUs identified in both groups (Fig. 1A).

For alpha diversity, we used four indices including Evenness, Faith’s phylogenetic diversity (Faith’s pd), Observed-OTUs and Shannon to evaluate the community richness and diversity of gut microbiome in the rectum. The Faith’s PD index was significantly lower in anal fistula patients than that in healthy individuals (Wilcoxon signed-rank test, *p-value* = 0.007) (Fig. 1B). Although the other three indices Evenness (Wilcoxon signed-rank test, *p-value* = 0.240), observed-OTUs (Wilcoxon signed-rank test, *p-value* = 0.240) and Shannon (Wilcoxon signed-rank test, *p-value* = 0.150) showed no significantly difference between two groups, the healthy individuals tended to have higher community richness and diversity of gut microbiome in the rectum than anal fistula patients (Fig. 1B).

For beta diversity, we also used four indices including Bray–Curtis distance, Jaccard distance, Unweighted Unifrac distance and Weighted Unifrac distance to further assess the similarities and differences between two groups. The principal coordinate analysis (PCoA) revealed a trend of separation between anal fistula patients and healthy individuals based on Jaccard distance (*p-value* = 0.002) and Unweighted Unifrac distance (*p-value* = 0.001) (Fig. 1C). Besides, the PCoA based on the Bray–Curtis distance (*p-value* = 0.098) and Weighted Unifrac distance (*p-value* = 0.159) showed no obvious differences between two groups (Fig. 1C). Taken together, the biodiversity of gut microbiome in the rectum revealed significant difference between anal fistula patients and healthy individuals.

### The composition of gut microbiome

**Fig. 2:**
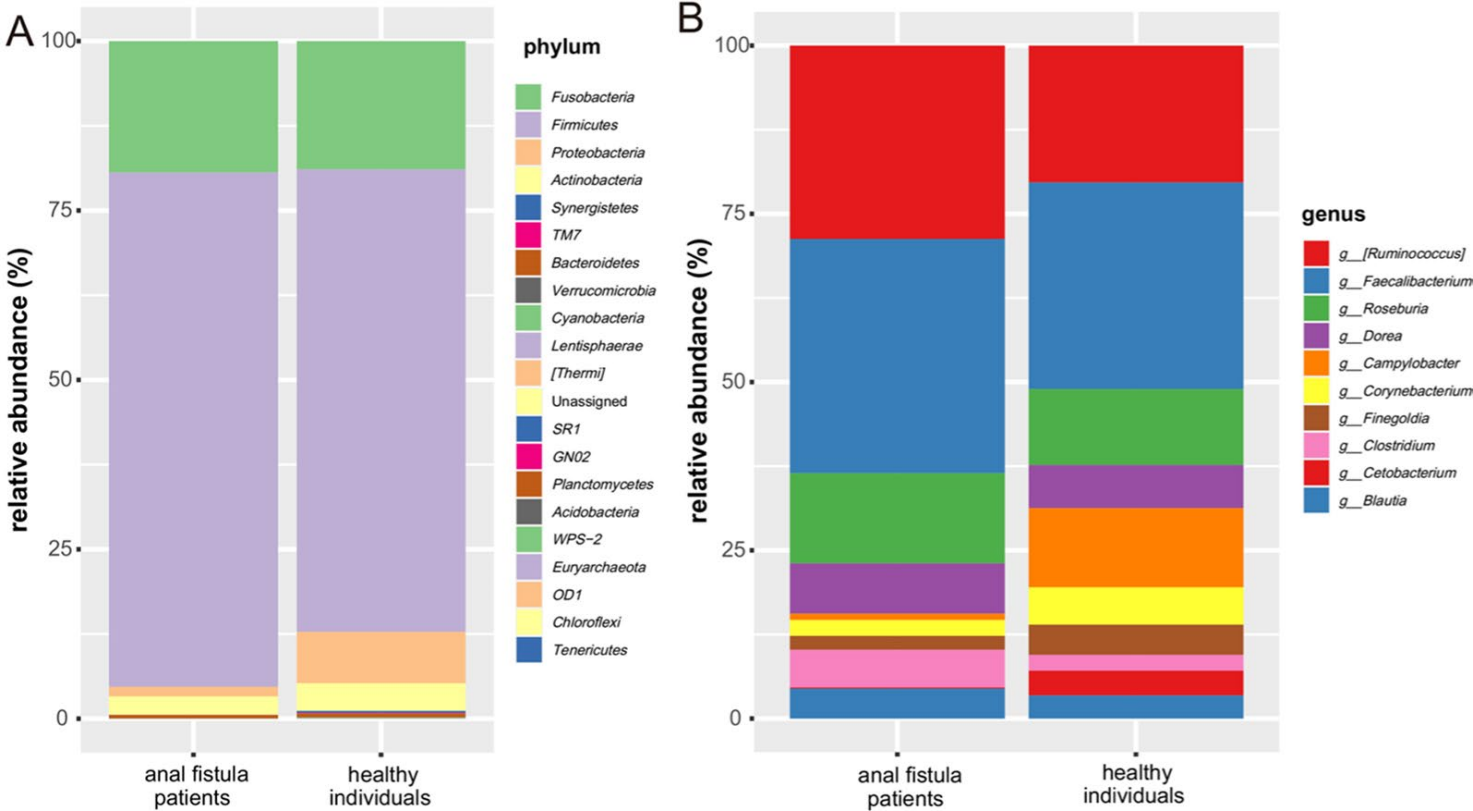
The composition of gut microbiome in the two groups. The barplots display the relative abundance of bacterial phyla (**A**) and the top 10 most dominant bacterial genus (**B**)

A total of 16 and 20 phyla were identified in the anal fistula patients and healthy individuals, respectively. *Firmicutes* was the dominant phylum in the two groups (75.92% and 68.24%), followed by *Fusobacteria* (19.37% and 18.94%) and *Proteobacteria* (1.41% and 7.59%) (Fig. 2A, Additional file 1: Fig. S1 and Table 2). Compared with the healthy individuals, the relative abundance of *Proteobacteria*, *Actinobacteria*, *Synergistetes*, *TM7* and *Thermi* were clearly decreased in the anal fistula patients (Table 2).

**Table 2:** Comparison of average relative abundance of gut microbiome between the anal fistula patients and healthy individuals

Taxa	Average relative abundance (%)		*P-*value
	Anal fistula patients	Healthy individuals	
Phylum			
*Fusobacteria*	19.373	18.937	0.558
*Firmicutes*	75.924	68.245	0.272
*Proteobacteria*	1.415	7.588	0.000
*Actinobacteria*	2.708	4.072	0.007
*Synergistetes*	0.030	0.279	0.000
*TM7*	0.004	0.100	0.002
*Bacteroidetes*	0.490	0.566	0.436
*Verrucomicrobia*	0.020	0.058	0.112
*Cyanobacteria*	0.015	0.087	0.370
*Lentisphaerae*	0.014	0.043	0.078
*Thermi*	0	0.009	0.002
*Unassigned*	0.004	0.006	0.989
*SR1*	0	0.006	0.089
*GN02*	0	0.001	0.337
*Planctomycetes*	0.001	0	0.317
*Acidobacteria*	0.001	0.0002	0.528
*WPS-2*	0	0.001	0.337
*Euryarchaeota*	0	0.001	0.337
*OD1*	0.001	0.001	0.730
*Chloroflexi*	0.001	0.0004	0.975
*Tenericutes*	0.001	0.001	0.202
Genus			
*Ruminococcus*	28.730	20.330	0.200
*Faecalibacterium*	34.810	30.690	0.365
*Roseburia*	13.400	11.300	0.876
*Dorea*	7.470	6.400	0.636
*Campylobacter*	0.950	11.750	0.002
*Corynebacterium*	2.350	5.560	0.000
*Finegoldia*	2.060	4.490	0.010
*Clostridium*	5.610	2.360	0.000
*Cetobacterium*	0.160	3.680	0.064
*Blautia*	4.460	3.430	0.428

At the genus level, *Faecalibacterium* (34.81%), *Ruminococcus* (28.73%), *Roseburia* (13.4%) and *Dorea* (7.47%) were the dominant bacterial genus in the anal fistula patients. In the healthy individuals, *Faecalibacterium* (30.69%), *Ruminococcus* (20.33%), *Campylobacter* (11.75%) and *Roseburia* (11.3%) were the major genera (Fig. 2B, Additional file 1: Fig. S1 and Table 2). We found that the relative abundance of *Clostridium* was clearly increased, while the *Campylobacter*, *Corynebacterium* and *Finegoldia* was decreased significantly in the anal fistula patients (Table 2).

### Differential microbiota of gut microbiome

**Fig. 3:**
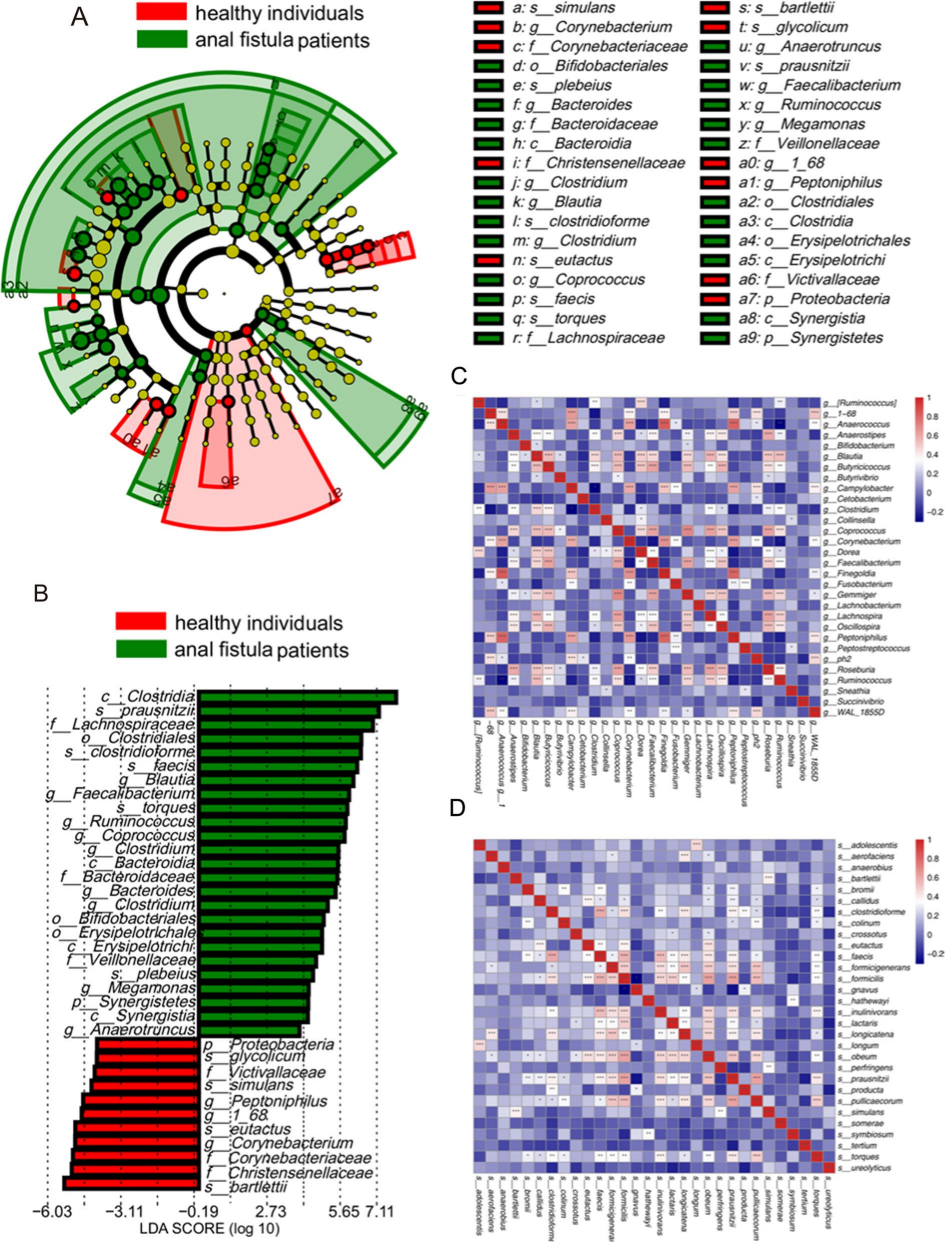
Differential microbiota of gut microbiome between anal fistula patients and healthy individuals. **A** The cladogram displays the taxonomic hierarchical structure of 36 discriminative biomarkers identified by the LEfSe analysis. **B** Barplots show the LDA score of 36 discriminative biomarkers. The color of bars represents the values of log10(LDA). The heatmap displays the spearman correlations among the top 30 most abundant genera (**C**) and species (**D**). *p-value < 0.05, ** *p-value* < 0.01, *** *p-value* < 0.001

We used the LEfSe analysis to identify the differential microbiota of gut microbiome in the rectum between anal fistula patients and healthy individuals. A total of 36 discriminative biomarkers were identified with LDA score > 4 and *p-value* < 0.05 between two groups (Fig. 3A). At the phylum level, *Synergistetes* was enriched in anal fistula patients, while *Proteobacteria* was higher in healthy individuals. At the genus level, the gut microbiome in the rectum of anal fistula patients was highly enriched with *Blautia*, *Faecalibacterium*, *Ruminococcus*, *Coprococcus*, *Bacteroides*, *Clostridium*, *Megamonas* and *Anaerotruncus*, while the microbiome of healthy individuals was enriched with *Peptoniphilus* and *Corynebacterium*. At the species level, *prausnitzii*, *clostridioforme*, *faecis*, *torques* and *plebeius* were enriched significantly in anal fistula patients, while *glycolicum*, *simulans*, *eutactus* and *bartlettii* were enriched in healthy individuals (Fig. 3B).

In order to verify the association between gut microbiomes, we calculated the Spearman correlations among the top 30 most abundant genera and species (Fig. 3C and D). We found that *Faecalibacterium*, both an enriched significantly in anal fistula patients and the most dominant genus, was positively correlated with *Butyricicoccus*, *Coprococcus* and *Gemmiger*. The combined effect of these microbiota may influence the progression of anal fistula.

### Establishing and evaluating the diagnostic prediction model

**Fig. 4:**
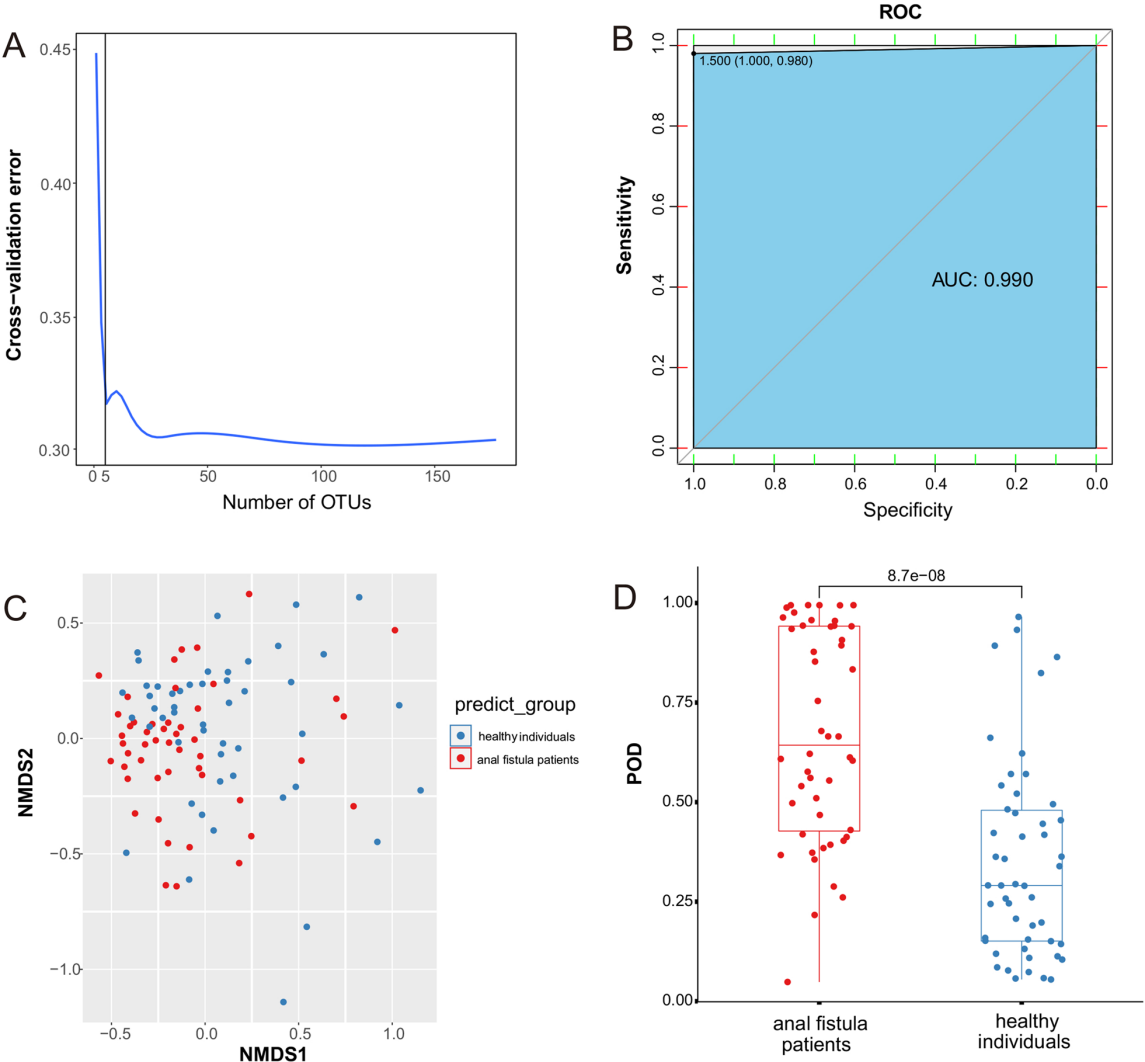
Identification of bacterial taxa related diagnostic biomarkers for anal fistula patients. **A** Ten trials of tenfold cross-validation indicate that the optimal biomarkers are 10. **B** ROC curves of the prediction model. **C** PCoA plot represents the predicted results of diagnostic prediction model. **D** The barplots show the values of POD between anal fistula patients and healthy individuals

Based on the relative abundance of gut microbiome, we applied the random forest model to establish the diagnostic prediction model. In order to enhance the accuracy and avoid overfitting, we used ten trials of tenfold cross-validation to determine the optimal biomarkers. Then, 5 microbiota of gut microbiome were selected to construct the prediction model (Fig. 4A and Additional file 1: Table S2). As shown in Fig. 4B, the area under curve (AUC) reached 0.990 between anal fistula patients and healthy individuals, indicating high accuracy of the prediction model. This diagnostic prediction model can distinguish well between patients and healthy individuals (Fig. 4C). Finally, we compared the values of POD between two groups, the POD of anal fistula patients was significantly higher than that in healthy individuals (Fig. 4D). The above results suggested that the diagnostic prediction model based on the microbiota of gut microbiome has high accuracy and may provide innovative biomarkers for anal fistula patients.

## Discussion

Anal fistula is a common proctological disease, but the thorough mechanisms of the anal fistula formation are still unclear. Nowadays, there is a widely accepted doctrine of the cryptoglandular theory, but there are many debatable points (Gosselink et al. 2015; Rizzo et al. 2010). Firstly, many patients with abscess do not lead to the anal fistula (Hamadani et al. 2009). Secondly, Mitalas examined the fistula tract of 53 patients and no mucin-producing cells were detected in the anal gland tissue of all patients (Mitalas et al. 2012). An increasing number of studies have shown that the disruption of the balance of the gut microbiome can lead to the intestinal diseases like colorectal cancer (Brennan and Garrett 2016) and inflammatory bowel diseases (Frank et al. 2007). We hypothesize that the disruption of the balance of the gut microbiome plays an important role in the pathogenesis of anal fistula. Here, we used the 16S rRNA gene sequencing to profile the intestinal microbiome between anal fistula patients and healthy individuals. Notably, there were significant differences in distribution of microbiota between the groups.

For alpha diversity and beta diversity, the Faith’s PD index was significantly lower in anal fistula patients than that in healthy individuals. The other three indices Evenness, observed-OTUs and Shannon also showed that the healthy individuals tended to have higher community richness and diversity of gut microbiome in the rectum than anal fistula patients. The same results were also seen in the analysis of beta diversity. Above all, the biodiversity of gut microbiome in the rectum revealed significant difference between anal fistula patients and healthy individuals.

The composition of the dominant bacteria was quite similar between the two groups, but the relative abundance was different. For example, at the phylum level, *Synergistetes* was enriched in anal fistula patients, while *Proteobacteria* was higher in healthy individuals. The phylum *Synergistetes*, a gram-negative anaerobe in the oral cavity, was related to periodontal and endodontic infections (Baumgartner et al. 2012). In addition, some studies found that the infection of *Synergistetes* was associated with the genital tract like pelvic abscesses (Jumas-Bilak et al. 2007; Marchandin et al. 2010). We believe that the increase in the relative abundance of *Synergistetes* may cause persistent inflammation, which can lead to the anal fistula.

Previous research indicates that the detrimental strains of *Proteobacteria* was increased in most diseases (Breton et al. 2022). Interestingly in this study, this bacterium was decreased in the patients with anal fistula, and the same trend was also found in another article (Qiu et al. 2022). We speculated that *Proteobacteria* may a protective factor for the anal fistula, just like the infection of *Helicobacter pylori* is a protective factor for Crohn´s disease (Wang and Xu 2019).

We also found that at the genus level, *Blautia*, *Faecalibacterium*, *Ruminococcus*, *Coprococcus*, *Bacteroides*, *Clostridium*, *Megamonas* and *Anaerotruncus* were highly enriched in anal fistula patients, while the microbiome of healthy individuals was enriched with *Peptoniphilus* and *Corynebacterium*. These gut microbiomes had been reported in other intestinal diseases (Brown et al. 2019; Forbes et al. 2018; Li et al. 2016; Sanders et al. 2019; Schirmer et al. 2019; Wang et al. 2019). For example, a study about inflammatory bowel diseases found that *Blautia*, *Faecalibacterium* and *Ruminococcus* were considered as the important taxa in Crohn´s disease and ulcerative colitis. Moreover, the disruption of *Ruminococcus* was correlated with the poor response to anti-TNFα therapy of patients with Crohn´s disease (Schirmer et al. 2019). Besides, the *Megamonas* genus has been reported to be associated with the inflammation (Ling et al. 2016; Liu et al. 2021).

We calculated the Spearman correlations among the different genera and species, which can reveal the interactions between these taxa. The genus *Faecalibacterium* was positively correlated with *Butyricicoccus*, *Coprococcus* and *Gemmiger*, indicating that these bacteria may support each other’s growth (Hong et al. 2022). What’s more, the positive correlation between *Faecalibacterium* and *Butyricicoccus* was also reported to change the intestinal permeability in older adults (Peron et al. 2021). We believe that this combined effect may influence the progression of anal fistula. Besides, 5 microbiota of gut microbiome were selected to construct the prediction model, and the AUC reached 0.990. The POD of anal fistula patients was significantly higher than that in healthy individuals. The above results suggested that the diagnostic prediction model based on the microbiota of gut microbiome has high accuracy and may provide innovative biomarkers for anal fistula patients.

Although this study explores the distinct gut microbiome of rectum in anal fistula patients, there are several limitations that need to be considered. Firstly, our research only describes the phenomenon and lacks direct experimental verification. Second, the taxa below the genus level may not be very clear by 16S rRNA gene sequencing. The application of metagenomics will allow us to gain a clearer understanding of microbial taxa. Thirdly, we collected the microbiome samples extracted by repeatedly wiping the rectal wall with intestinal swab, rather than fecal samples. The samples generated by this workflow may not be representative of the luminal microbial population. But, after the irrigation, the remain samples represent those microbiomes that are more stable and more important for diseases. Despite these drawbacks, we also provide important clues for analyzing gut microbiome of rectum in anal fistula patient.

## Supplementary Information

**Additional file 1.** **Table S1.** Sequencing information summary. **Table S2.** List of the 5 biomarkers in the optimal marker set. **Figure S1.** Bacterial abundance and distribution in each sample.

## Data Availability

The link of gut microbiome data is: accession PRJEB59219.
