# Supplementary material for: The potential roles of gut microbiome in anal fistula
Source: AMB Express. 2023 Jun 10;13:58. doi: 10.1186/s13568-023-01560-9 (PMC10257611; doi:10.1186/s13568-023-01560-9)
Supplement: Supplementary file 1 — Additional file 1. Table S1. Sequencing information summary. Table S2. List of the 5 biomarkers in the optimal marker set. Figure S1. Bacterial abundance and distribution in each sample. [file 13568_2023_1560_MOESM1_ESM.pdf]

Table S1. Sequencing information summary: number of raw reads, passed filtered reads, and after denoised reads and percentage of passed filtered reads and after denoised reads.

Sample-id	Group	Raw reads	Passed filtered reads	After denoised reads
S20210127-2629	Patient	219100	192293(87.76)	189714(86.59)
S20210127-2630	Patient	151276	132846(87.82)	130067(85.98)
S20210127-2631	Patient	181439	155462(85.68)	152929(84.29)
S20210127-2632	Patient	141146	123739(87.67)	119536(84.69)
S20210127-2633	Patient	163585	138964(84.95)	136976(83.73)
S20210127-2634	Patient	189124	166091(87.82)	163867(86.65)
S20210127-2635	Patient	153569	134435(87.54)	132036(85.98)
S20210127-2636	Patient	181916	159718(87.8)	156784(86.18)
S20210127-2637	Patient	200107	175504(87.71)	173766(86.84)
S20210127-2638	Healthy	188337	165833(88.05)	161790(85.90)
S20210127-2639	Healthy	183163	160374(87.56)	157942(86.23)
S20210127-2640	Healthy	139455	121772(87.32)	119153(85.44)
S20210127-2641	Healthy	194648	170772(87.73)	167777(86.20)
S20210127-2642	Healthy	236696	207029(87.47)	199203(84.16)
S20210127-2643	Healthy	198546	175209(88.25)	170893(86.07)
S20210127-2644	Healthy	183828	161409(87.8)	157883(85.89)
S20210127-2645	Healthy	197639	170586(86.31)	168081(85.04)
S20210127-2646	Healthy	184768	162559(87.98)	158855(85.98)
S20210127-2647	Healthy	224600	188881(84.1)	186454(83.02)
S20210127-2648	Healthy	164115	143950(87.71)	138527(84.41)
S20210127-2649	Healthy	169142	146732(86.75)	144782(85.60)
S20210127-2650	Healthy	258740	227739(88.02)	224336(86.70)
S20210127-2651	Healthy	171590	149357(87.04)	146731(85.51)
S20210127-2652	Healthy	132361	115946(87.6)	113647(85.86)
S20210127-2653	Healthy	184320	162135(87.96)	159011(86.27)
S20210127-2654	Healthy	159979	140086(87.57)	137243(85.79)
S20210127-2655	Healthy	123446	107419(87.02)	105473(85.44)
S20210127-2656	Healthy	184836	161527(87.39)	158758(85.89)
S20210127-2657	Healthy	138656	123021(88.72)	121837(87.87)
S20210127-2658	Healthy	145222	127417(87.74)	124258(85.56)
S20210127-2659	Healthy	221011	194218(87.88)	190298(86.10)
S20210127-2660	Healthy	189358	166161(87.75)	161813(85.45)
S20210127-2661	Patient	124001	109556(88.35)	108095(87.17)
S20210127-2662	Patient	169737	148715(87.61)	146530(86.33)
S20210127-2663	Patient	212313	187122(88.13)	185169(87.22)
S20210127-2664	Patient	207812	178508(85.9)	175376(84.39)
S20210127-2665	Patient	187508	163497(87.19)	161121(85.93)
S20210127-2666	Patient	190654	165297(86.7)	162710(85.34)
S20210127-2667	Patient	173823	152874(87.95)	150529(86.60)
S20210127-2668	Patient	227436	197000(86.62)	194382(85.47)
S20210127-2669	Patient	142646	124521(87.29)	123244(86.40)

S20210127-2670	Patient	193795	169194(87.31)	166269(85.80)
S20210127-2671	Patient	271501	238079(87.69)	235435(86.72)
S20210127-2672	Patient	154763	136367(88.11)	133820(86.47)
S20210127-2673	Patient	120422	105661(87.74)	102356(85.00)
S20210127-2674	Patient	198143	172544(87.08)	170695(86.15)
S20210127-2675	Patient	227676	197948(86.94)	195091(85.69)
S20210127-2676	Patient	191300	166794(87.19)	164181(85.82)
S20210127-2677	Patient	207535	181614(87.51)	178372(85.95)
S20210127-2678	Patient	236238	208184(88.12)	206477(87.40)
S20210127-2679	Patient	240163	206210(85.86)	203963(84.93)
S20210127-2680	Patient	176781	152019(85.99)	149913(84.80)
S20210127-2681	Patient	227348	186984(82.25)	183862(80.87)
S20210127-2682	Patient	143262	125785(87.8)	122867(85.76)
S20210127-2684	Patient	215989	190376(88.14)	186718(86.45)
S20210127-2685	Patient	118285	103691(87.66)	101653(85.94)
S20210127-2686	Patient	96524	83407(86.41)	82094(85.05)
S20210127-2687	Patient	147916	128178(86.66)	126346(85.42)
S20210127-2688	Patient	188112	162841(86.57)	160413(85.28)
S20210127-2689	Patient	91281	79208(86.77)	76036(83.30)
S20210127-2690	Patient	167326	145957(87.23)	143938(86.02)
S20210127-2691	Patient	132094	115660(87.56)	113503(85.93)
S20210127-2692	Patient	162279	141223(87.02)	139585(86.02)
S20210127-2693	Patient	102236	89489(87.53)	87619(85.70)
S20210127-2694	Patient	213158	185825(87.18)	182781(85.75)
S20210127-2695	Patient	95940	84917(88.51)	82736(86.24)
S20210127-2696	Patient	183518	161135(87.8)	158779(86.52)
S20210127-2697	Patient	151010	130553(86.45)	128232(84.92)
S20210127-2698	Patient	175206	151262(86.33)	148424(84.71)
S20210127-2699	Patient	130783	113945(87.13)	112447(85.98)
S20210127-2700	Patient	142958	122535(85.71)	120155(84.05)
S20210127-2701	Healthy	154746	135518(87.57)	132222(85.44)
S20210127-2702	Healthy	149350	129838(86.94)	126384(84.62)
S20210127-2703	Healthy	104515	91568(87.61)	90163(86.27)
S20210127-2704	Healthy	148092	130020(87.8)	127775(86.28)
S20210127-2705	Healthy	220191	193897(88.06)	191246(86.85)
S20210127-2706	Healthy	201409	176407(87.59)	173036(85.91)
S20210127-2707	Healthy	159183	138522(87.02)	136325(85.64)
S20210127-2708	Healthy	178717	152363(85.25)	151092(84.54)
S20210127-2709	Healthy	192991	168462(87.29)	164736(85.36)
S20210127-2710	Healthy	174467	152629(87.48)	148367(85.04)
S20210127-2711	Healthy	135081	118347(87.61)	116554(86.28)
S20210127-2712	Healthy	168884	147419(87.29)	145720(86.28)
S20210127-2713	Healthy	194643	170567(87.63)	166720(85.65)
S20210127-2714	Healthy	127044	110091(86.66)	108222(85.18)

S20210127-2715	Healthy	151870	133047(87.61)	131247(86.42)
S20210127-2716	Healthy	179246	157551(87.9)	152632(85.15)
S20210127-2717	Healthy	150661	131519(87.29)	129279(85.81)
S20210127-2718	Healthy	156977	137038(87.3)	130508(83.14)
S20210127-2719	Healthy	80452	70356(87.45)	67728(84.18)
S20210127-2720	Healthy	123530	108508(87.84)	107425(86.96)
S20210127-2721	Healthy	90557	75765(83.67)	74162(81.90)
S20210127-2722	Healthy	174735	153788(88.01)	150098(85.90)
S20210127-2723	Healthy	133674	117327(87.77)	113769(85.11)
S20210127-2724	Healthy	135612	118943(87.71)	116693(86.05)
S20210127-2725	Healthy	141911	124284(87.58)	122027(85.99)
S20210127-2726	Healthy	185759	163886(88.23)	161738(87.07)
S20210127-2727	Healthy	80297	70128(87.34)	67179(83.66)

Table S2. List of the 5 biomarkers in the optimal marker set

s__clostridioforme
s__bartlettii
g__Corynebacterium
s__orale
g__Anaerotruncus

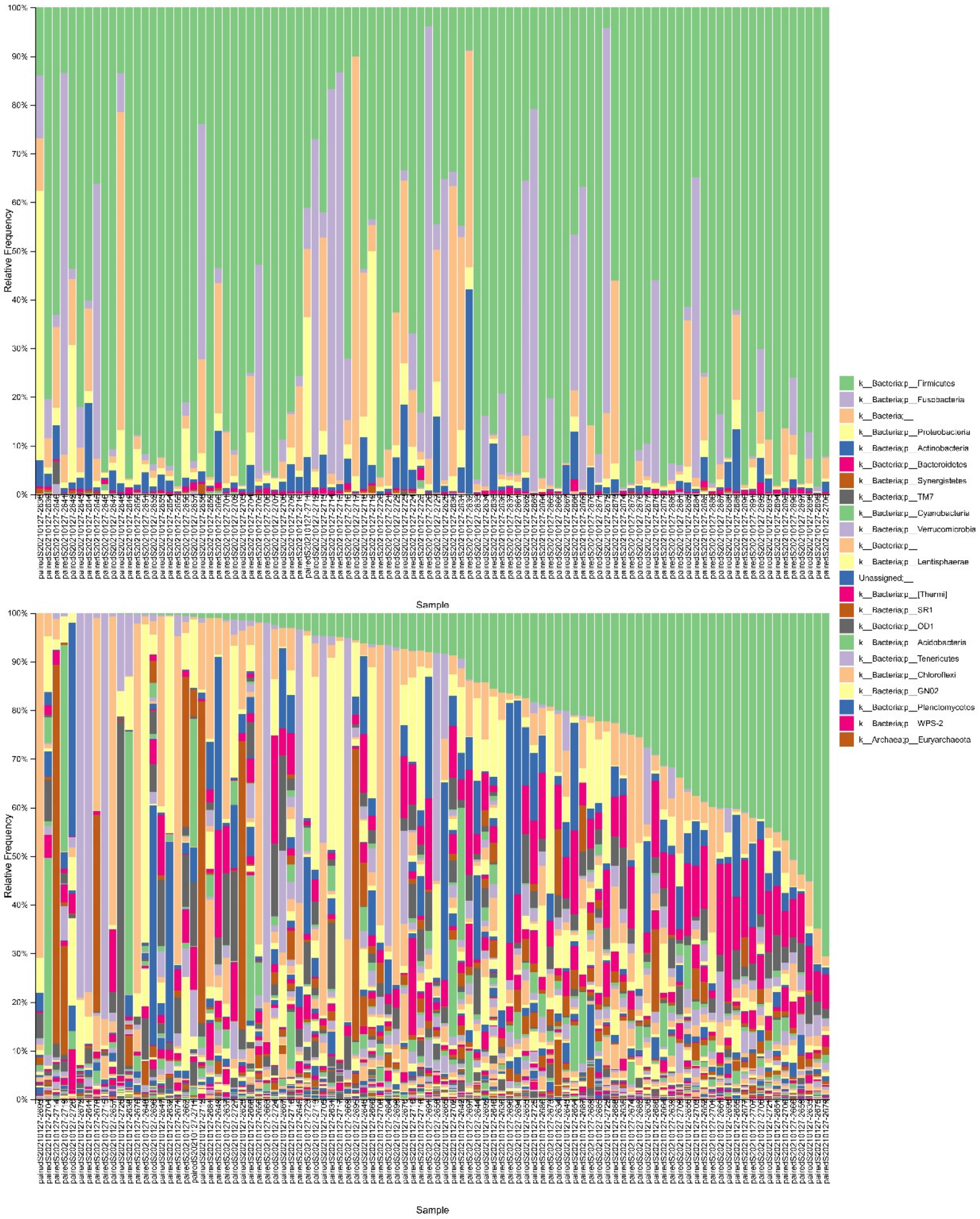

Figure S1. Bacterial abundance and distribution in each sample.
